# Molecular Characterization of *Borrelia persica*, the Agent of Tick Borne Relapsing Fever in Israel and the Palestinian Authority

**DOI:** 10.1371/journal.pone.0014105

**Published:** 2010-11-24

**Authors:** Gracia Safdie, Iba Y. Farrah, Reem Yahia, Esther Marva, Amos Wilamowski, Samer S. Sawalha, Naama Wald, Judith Schmiedel, Annette Moter, Ulf B. Göbel, Herve Bercovier, Ziad Abdeen, Marc V. Assous, Yolanta Fishman

**Affiliations:** 1 Department of Molecular Genetics and Microbiology, Faculty of Medicine, Hebrew University of Jerusalem, Jerusalem, Israel; 2 The Ministry of Health, Central State Laboratories, Jerusalem, Israel; 3 Department of Primary Health Care, Palestinian Ministry of Health, Ramallah; 4 Department of Microbiology and Hygiene, Charite University Medicine-Berlin, Berlin, Germany; 5 Department of Community Health, Medical School, Al Quds University, Jerusalem, Israel; 6 Laboratory of Microbiology and Immunology, Shaare Zedek Medical Center, Jerusalem, Israel; East Carolina University School of Medicine, United States of America

## Abstract

The identification of the Tick Borne Relapsing Fever (TBRF) agent in Israel and the Palestinian Authority relies on the morphology and the association of *Borrelia persica* with its vector *Ornithodoros tholozani*. Molecular based data on *B. persica* are very scarce as the organism is still non-cultivable. In this study, we were able to sequence three complete 16S rRNA genes, 12 partial *fla*B genes, 18 partial *glp*Q genes, 16 *rrs-ile*T intergenic spacers (IGS) from nine ticks and ten human blood samples originating from the West Bank and Israel. In one sample we sequenced 7231 contiguous base pairs that covered completely the region from the 5′end of the 16S rRNA gene to the 5′end of the 23S rRNA gene comprising the whole 16S rRNA (*rrs*), and the following genes: Ala tRNA (*ala*T), Ile tRNA *(ile*T), adenylosuccinate lyase *(pur*B), adenylosuccinate synthetase (*pur*A), methylpurine-DNA glycosylase (*mag*), hypoxanthine-guanine phosphoribosyltransferase (*hp*t), an hydrolase (HAD superfamily) and a 135 bp 5′ fragment of the 23S rRNA (*rrl*A) genes. Phylogenic sequence analysis defined all the *Borrelia* isolates from *O. tholozani* and from human TBRF cases in Israel and the West Bank as *B. persica* that clustered between the African and the New World TBRF species. Gene organization of the intergenic spacer between the 16S rRNA and the 23S rRNA was similar to that of other TBRF *Borrelia* species and different from the Lyme disease *Borrelia* species. Variants of *B. persica* were found among the different genes of the different isolates even in the same sampling area.

## Introduction

Tick Borne Relapsing Fever (TBRF) is characterized by recurring fever attacks, usually of decreasing intensity and accompanied by nonspecific symptoms like myalgia, headache and gastrointestinal symptoms. It is caused by a dozen different spirochetes species of the genus *Borrelia* that are endemic in different geographical areas [1]. Each *Borrelia* species is transmitted within a geographical range by a specific species of soft ticks of the genus *Ornithodoros* (Argasidae) [2]. In Israel and in the Palestinian Authority the main agent of TBRF is described in the literature as *Borrelia persica* transmitted by the cave tick *Ornithodoros tholozani* [3], [4], [5]. However, because *B. persica* has not been cultured yet “in vitro”, its characterization is mainly based on the geographic distribution of TBRF cases, the clinical and epidemiological setting, its morphology and finally on the presence of the vector *O. tholozani* as a source of the transmission. Accordingly the distribution of *B. persica* covers Central Asia (Iran, Kazakhstan, Kyrgyzstan, Tajikistan, Turkmenistan, Uzbekistan, Afghanistan and India) [3] and in the Middle East (Iraq, Jordan, Syria, Israel, and Egypt) [3].

TBRF is a rare disease in Israel but probably under-reported partly due to the fact that the disease is relatively mild and usually without a serious outcome [3]. The diagnosis and presence of TBRF in the Palestinian Authority is even less studied although geographical conditions suggest that the disease and the bacterium-host pair (*B. persic*a-*O. tholozani*) are also present. Indeed, cases recorded in Jordan between 1959 and 1969 included cases from the West Bank of the River Jordan [6].

Molecular data on *B. persica* are very scarce and limited to a single complete sequence of the 16S ribosomal RNA (rRNA) gene (*rrs*) of a Persian strain [7] and partial sequences of the 16S rRNA (*rrs*), of the flagellin (*fla*B) [2] and the glycerophosphodiester phosphodiesterase (*glp*Q) genes [4].

A thorough molecular study is the prerequisite for the development of molecular assays for characterization of the *Borrelia* species involved in relapsing fever in Israel and the Palestinian Authority and consequently for the development of specific epidemiological and diagnostic assays. In this study, we sequenced three complete 16S rRNA genes, 12 partial *fla*B genes, 18 partial *glp*Q genes, 16 *rrs-ile*T intergenic spacers (IGS) from nine ticks and ten human blood samples originating from the West Bank and Israel. In one sample we sequenced 7231 contiguous base pairs that covered completely the region from the 5′ end of the 16S rRNA gene (*rrs*) to the 5′end of the 23S rRNA gene (*rrl*A). Using this information we were able to characterize by molecular methods the etiological agent of TBRF in Israel and the Palestinian Authorities present in *O. tholozani* and in human samples and demonstrate that it is very close if not identical to the reference stain of *B. persica* [7] thus providing definite evidence that *B. persica* is the main agent of TBRF in the Palestinian Authority and in Israel.

## Materials and Methods

### Ethics Statement

The study was considered as part of a routine program for TBRF diagnosis that could improve this diagnosis for future cases of TBFR and its control. The Helsinki committee of Shaare Tzedek ruled that in this particular case, formal IRB approval and written consent from patients are not required given that medical care would not be modified by the research process, given the retrospective nature of the research and that the research did not involve any procedures for which written consent is normally required outside of the research context and because the study used samples that were routinely collected for use in approved routine tests (Waiver p 27/10). All samples were anonymously obtained, no human experimentation was conducted and no human genetic study was performed.

### Samples

The TBRF diagnosis in patients was established as previously reported [8]. Samples of human blood were sent to the Parasitology Reference Center (Ministry of Health, Jerusalem) and to Israeli hospitals for TBRF diagnosis. Fresh human blood samples were examined by dark field microscopy for viable *Borrelia* and frozen at −80°C for molecular studies.

Collection of ticks in the West Bank was conducted by Al Quds University with the collaboration of the Palestinian Ministry of Health using CO_2_ traps as described previously [2]. Specimens of ticks were collected from different caves in the West Bank and were preserved in 70% alcohol. Ticks were phenotypically identified as *O. tholozani* by the Entomology Laboratory Israeli Ministry of Health, Jerusalem or by the Palestinian Ministry of Health, Ramallah. The list and location of the samples investigated are given in Table 1.

**Table 1:** Characterization of the isolates investigated in this work.

Host	Isolate	Geographic	*fla*B^1^	*glp*Q	*rrs*	*rrs-ile*T	*pur*A	*rrs-rrl*A
		location				IGS		IGS
			Types	Genovars				
Human	H1015	Merav (Gallilee)	I	G1	R2	a	P1	partial
	H1039	Merav (Galliee)	I	G3	R1	a	nd	partial
	H1042	Israel	II	G3	R1	b	nd	partial
	H1201	Safed	II	G2	R1	a	P1	partial
	H1254	Lod	II	nd^2^	R1	nd	nd	partial
	H1369	Arad	II	G4	R2	a	P4	partial
	H1370	Arad	I	G3	R1	b	P1	partial
	H1374	Safed	I	G1	R1	b	P3	partial
	HL2610	Jerusalem	II	G2	R2	a	P3	**Complete**
	HS3011	Jerusalem	I	G2	R2	b	P2	partial
*O. tholozani*	TGd1	Lod (Gizmo)	I	G1	nd	c	P1	Partial
	TG52	Lod (Gimzo)	II	G2	nd	nd	nd	nd
	T4111.1	Bethlehem	nd	G2	nd	nd	nd	nd
	T241.4	Tubas	nd	G2	nd	b	nd	partial
	T241.9	Tubas	nd	G2	nd	b	nd	partial
	T241.11	Tubas	nd	G2	nd	b	nd	partial
	T711.1	Ramallah	I	G2	nd	b	nd	partial
	T711.9	Ramallah	I	G2	nd	b	nd	partial
	T711.11	Ramallah	nd	G2	nd	b	nd	partial

1 Typing and genovar determination were performed for the genes encoding Flagellin (*fla*B), Glycerophosphodiester phosphodiesterase (*glp*Q), 16S rRNA (rrs), Adenylosuccinate synthetase (*pur*A), and the intergenic spacer (IGS) between *rrs* and the ile tRNA (*ile*T) and between *rrs* and the 23S rRNA (*rrlA*).

2 nd: not done.

### DNA extraction and screening for the presence of *Borrelia* species

Total DNA was extracted from individual ticks or from blood samples obtained from infected patients as described by Assous et al. [2]. DNeasy blood & tissue purification kit (Qiagen, Hilden, Germany) was used for DNA extractions as recommended by the manufacturer.

Preliminary screening for the presence of *Borrelia* in the samples was performed by amplifying a 750 bp partial fragment of the flagellin gene (*fla*B) by a polymerase chain reaction (PCR) with primers BOR1 and BOR2 as described by Assous et al. [2]. Details of all primers used in this work are listed in Table 2. Two negative controls, water as well as DNA extracted from uninfected ticks were included in each run. Amplified DNA was purified using the Qiaquick PCR purification kit (Qiagen, Hilden Germany) or ExoSAP-IT (USB, Cleveland, USA) as recommended by the manufacturers and used for sequencing (Hylabs, Rehovot, Israel). The amplified fragments were sequenced directly with primers used for the amplification reaction or after cloning in the EcoRV site of plasmid pBluescript using the T7 and T3 universal primers. In all cases both strands of each fragment were sequenced. Sequences were analyzed with the Vector NTI advance 11 software (Invitrogen, UK).

**Table 2:** List of the primers used in this work.

Target locus	primer name^1^	Primer sequence 5′-3′	reference
*rrs*	16s5′	AGT TTG ATC CTG GCT TAG AAC	This work
	16s1F	GAA GGT CGA AAG ATT GTA AAG	This work
	16s 2F	TAG GAA ATG ACA AGG TGA TGA CG	This work
	16s3R	CGT CAT CAC CTT GTC ATT TC	This work
	16s4R	ACG CAT AGA CTT GCA TAT CC	This work
	16s6F	GAT TAG ATA CCC TGG TAG	This work
	16s7R	CCT TTG AGT TTC ACT CTT G	This work
	16s8F	CAC AAG CGG TGG AGC ATG TG	This work
	16s9F	GAT GAC GTC AAA TCA TCA TGG	This work
	16s10R	CCA TGA TGA TTT GAC GTC ATC	This work
	16s11R	TAC GAT TAC TAG CGA TTC CAA C	This work
	16s3′	TGA TCC AGC CAC ACT	This work
*rrs-ile*T IGS	IGSaF	GTA TGT TTA GTG AGG GGG GTG	[8]
	IGSaFn	AGG GGG GTG AAG TCG TAA CAA G	[8]
	IGSaR	GGA TCA TAG CTC AGG TGG TTA G	[8]
	IGSaRn	GTC TGA TAA ACC TGA GGT CGG A	[8]
*rrl*A*-ile*T IGS	IGSb1F	GCA TTG AGC TTG AGC TTT GCT C	This work
	IGSb1Fn	TAT TTG ATT ATT GCT TAG ATG GAC C	This work
	IGSb1R	GCT AGA GAA TAT ATT GAA TTT ATA G	This work
	IGSb1Rn	TTT AGG AAT CAA GAG TGG ATC	This work
	IGSb11Fs	ATA GGT TAA ATG TCG TGG CAT CTG	This work
	IGSb12Rs	GCA TAC TCT TCA CCT GAA TAA TC	This work
	IGSb13Rs	GCA ATC TAG CTC AAA GTG CAC	This work
	IGSb2F	ATG CAC TTG TAG TAC CAA GAT G	This work
	IGSb2R	CGA CCA CAT TGG AGA TGA AAT TAG	This work
	IGSb3F	CTA TAA ATT CAA TAT ATT CTC TAG C	This work
	IGSb3R	ATG GCA ATT TAT GCA GTT GTT GG	This work
	IGSb4F	AAC ACC TGA TGG TAA TAG	This work
	IGSb4Fn	CTA CAC TAG GTC CAA GTA TGC	This work
	IGSb4R	GTA CTT AGA TGG TTC ACT TCC CCT GG	This work
	IGSb4Rn	GGA TTA CTC CAT TCG GTA ATC TTG	This work
	IGSb41Rs	AAT CTT CTA CCA GTA GC	This work
	IGSb42Rs	TTG GAC TTC TAA TCT CTC ATC AG	This work
	IGSb43Fs	CCA AAT AAT GAG ATT ACA GCG	This work
*fla*B	BOR1	TAA TAC GTC AGC CAT AAA TGC	[2]
	BOR2	GCT CTT TGA TCA GTT ATC ATT	[2]
*glp*Q	glpQF	ATA GCT CAC AGA GGT GCA AGC GGA TAT TTA CCA GAA C	This work
	glpQR	ATC TTT TAC ATA TGA AGG CAA TGC ATC AAT TCT AAA	This work
	glpQRn	CAA TTC TAA ATG TAT AAG CAT GGA CTT TCA TGT TAT A	This work

1 An s in a primer's name indicates that it was used for sequencing only. Primers marked with n were used in nested PCR reactions.

### Amplification and sequencing of the complete 16S rRNA (*rrs*) gene

Overlapping fragments of the 16S ribosomal RNA gene (*rrs*) were amplified by PCR from the blood of patients using the following pairs of primers: 16S5′-16S4R, 16S1F-16S7R, 16S2F-16S10R, 16S6F-16S11R, and 16S8F-16S3′ (Table 2). One unit of Phusion DNA polymerase (Finnzymes, Keilaranta, Finland) was used for DNA amplification in a 50 µl reaction mixture containing buffer GC (supplied by the manufacturer). The PCR conditions were: initial denaturation at 98°C for 30 sec, followed by 30 amplification cycles (98°C for 10 sec, 56°C for 20 sec, 72°C for 30 sec), and a final extension step at 72°C for 6 min. A 5′ end fragment (16S5′-16S3R) and a 3′end fragment (16S9F-16S3′) were cloned in the EcoRV site of the plasmid pBluescript and sequenced using the T7 and T3 universal primers.

### Amplification and sequencing of the flagellin (*fla*B) gene

Determination of the flagellin type of different isolates was performed by sequencing a *fla*B fragment (750 bp) amplified using the genus-specific set of primers BOR1 and BOR2 (Table 2). For PCR amplification 1U BIOTAQ™ DNA polymerase (Bioline GMBH Germany) was used in a reaction mixture containing 1.6 mM MgCl_2_. Reaction conditions were as follows: initial denaturation step of 95°C for 1 min, followed by 40 cycles of 94°C for 30 sec, 57°C for 30 sec and 72°C for 30 sec with a final extension step of 72°C for 5 min.

### Amplification and sequencing of the glycerophosphodiester phosphodiesterase (*glp*Q) gene

After alignment of the *glp*Q gene sequences available in GenBank (see below), areas of high homology were identified and used to design primers glpqF and glpqR for the amplification and cloning of the *glp*Q gene from *B. persica* (Table 2). These primers delineate an 800 bp fragment covering 80% of the gene excluding the C- and N-termini. The PCR conditions were as described for the *rrs* gene. In the case of several tick isolates the *glp*Q fragment was not detected or was at the limit of detection after the first round of amplification; in these cases a nested protocol was applied using the same cycling conditions with an internal set of primers: glpqF and glpqRn (Table 2). The resulting fragments were sequenced and compared by phylogenetic analysis to *glp*Q genes sequences of *B. crocidurae* (AF247151), *B. recurrentis* (AF247155), *B. hermsii* (U40762), *B. parkeri* (AF247156), *B. turicatae* (AF247157) and *B. coriaceae* (AF247158).

### Sequencing of the *rrs*-*rrl*A spacer region

#### Sequencing the *rrs-ile*T intergenic spacer (IGS)

The *rrs-ile*T IGS portion of the *rrs*-*rrl*A spacer region was amplified using nested PCR with primers described by Bunikis et al. [9]. The first round of amplification was performed using Phusion DNA polymerase (Finnzymes, Keilaranta, Finland) and primers IGSaF and IGSaR (Table1). The following PCR conditions were used for these reactions: initial denaturation at 98°C for 30 sec followed by 35 cycles of 98°C for 10 sec, 56°C for 30 sec, 74°C for 30 sec and a final extension step of 72°C 10 min. This was followed by a nested reaction performed using the primers IGSaFn and IGSaRn (Table 2) and PCR conditions as described for *fla*B.

#### Sequencing of the *ile*T-*rrl*A region

By aligning sequences of intergenic regions of *Borrelia* species available in the GeneBank we were able to identify regions of homology that were used to design primers (Table 2) for amplification and sequencing of four overlapping fragments covering the entire region between the Ile tRNA and the 23S rRNA genes (Table 2). The fragment between *ile*T and *pur*A was amplified by a nested PCR reaction. Primers IGSb1F and IGSb1R were used in the first round and primers IGSb1Fn and IGSb1Rn were used in the nested reaction. Two overlapping fragments, a 1.3 kb fragment amplified with primers IGSb2F and IGSb2R and a 1.2 kb fragment amplified by primers IGSb3F and IGSb3R comprised the region between the *pur*A and the hydrolase genes. The fragment from the hydrolase to the *rrl*A gene was amplified by a nested reaction with primers IGSb4F and IGSb4R in the first round and IGSb4Fn and IGS4Rn for the nested reaction. PCR conditions were as described for the *rrs* gene. Sequencing was performed using the primers used for amplification. The sequence was completed by primer walking (Table 2).

### Phylogenic studies

The sequences obtained in this work were compared by phylogenic analysis to the sequences of *rrs*, *flab*, *glp*Q, *pur*A, *rrs-ile*T IGS and *rrs*- *rrl*A intergenic spacer from other TBRF *Borrelia* spp. with the following GenBank accession numbers: for *B. persica*: *rrs* (U42297, EU14141), DQ679904, *fla*B (DQ679910, DQ679911) and *glp*Q (EU914143); for *B. hispanica*: *rrs* (U42294) and the *rrs-ile*T IGS (FJ827590); for *B. crocidurae*: *rrs-ile*T IGS (DQ000287) and *glp*Q (AF247151); for *B.duttonii*: complete genome (CP000976), and *glp*Q (DQ346785 DQ909058); for *B. recurrentis*: complete genome (CP000993) and *rrs-ile*T IGS (DQ346784); for *B. hermsii*: complete genome (CP000048), *rrs* (U42292), *glp*Q (AY597707) and *rrs-ile*T IGS (DQ845749); for *B. turicatae*: complete genome (CP000049), *rrs* (U42299), *glp*Q (AY934642, AF247157) and *fla*B (AY934628); for *B. parkeri*: *rrs-ile*T IGS (DQ855550), *glp*Q (AY934635, AF247156) and for *B.bugdorferi*: *rrs* (AF477989).

Phylogenic analysis of the different loci was performed by using the MEGA software (version 4.1) [10] after multiple alignments of sequences by CLUSTAL W (1.83) [11]. Distance options were computed according to Maximum Composite Likelihood method [12]. Phylogenic trees were generated by Unweighted Pair-Group Method with Arithmetic averages (UPGMA) assuming that TBRF *Borrelia* species are ultrametric [13]. A bootstrap value of 250 replicates was taken to obtain more confidence in drawing parameters.

For the multilocus sequence analysis (MLSA), sequences from different loci (*rrs-ileT* IGS, *fla*B, *glp*Q, *pur*A) of seven *B. persica* isolates described in this work (six from human hosts: H1015, H1201, HL2610, HS3011, H1370, H1374 and one from tick: TGd1) were compared with homologue genes from other available TBRF *Borrelia* species. For each gene (*pur*A, *glp*Q, *fla*B and IGS), the orthologous nucleotide sequences from four *Borrelia* species (*B. recurrentis*, *B. duttonii*, *B. hermsii*, *B. turicatae*) and the *B. persica* isolates were aligned with CLUSTAL W (1.83) [11]. The alignments were then concatenated and the resulting long alignment was used to construct a phylogenetic tree. The tree was generated with the program PHYML (v2.4.5) [14]. Default values were used except for 100 bootstraps. The resulting tree was drawn using the Interactive Tree of Life web interface (http://itol.embl.de) [15].

### Nucleotide sequence accession numbers

Sequences determined in this study have been deposited in the GenBank and given the following accession numbers: *rrs* (HM161644-HM161653); *fla*B (HM194738-HM194743); *glp*Q (HM161654-HM161671); *rrs-ile*T IGS (HM194744-HM194759); *pur*A (HM194761-HM194767); *pur*B (HM194760); *mag* (HM194726-HM194728); *hpt* (HM194731-HM194737); Hydrolase (HM194729-HM194730); the 7231 bp contig containing the *rrs* gene and the *rrs*-*rrl*A intergenic spacer (HM131216).

## Results and Discussion

The failure to cultivate *in vitro B. persica* has prevented a comprehensive taxonomical study of this spirochete. When starting this work, available *B. persica* sequences were limited to one sequence of the 16S rRNA gene (*rrs*) [7] and fragments of the *fla*B [2] and *glp*Q genes [4]. To study the phylogenic relationship between Israeli and Palestinian TBRF Borrelia isolates and the *B. persica* prototype Iranian strain [7], we sequenced several loci from isolates collected in a variety of geographical location in the West Bank and in Israel from infected humans and from ticks.

### Analysis of the genomic region containing the 16S rRNA gene (*rrs*) and the entire intergenic spacer between the 16S rRNA gene and the 23S rRNA gene (*rrs-rrl*A)

**Figure 1:**
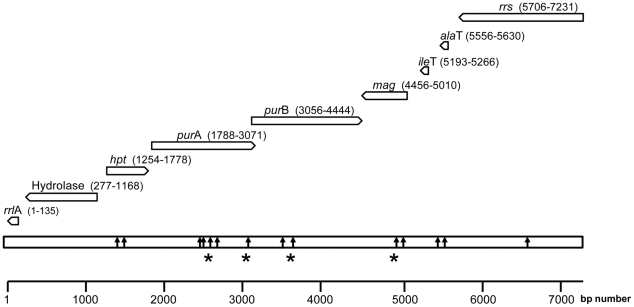
Physical map of the *rrs*-*rrl*A genomic region (7231 contig) of *Borrelia persica*. The genes and their orientation are indicated by empty arrows. The position of each locus on the 7231 contig is given in parentheses. Positions of nucleotides polymorphism are indicated by black arrows. Nucleotide changes resulting in amino acid modifications are marked with an asterisk.

**Figure 2:**
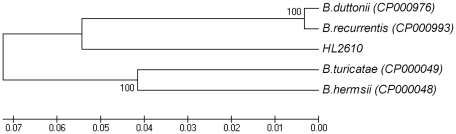
Phylogenetic tree based on the *rrs-rrl*A spacer region. The tree was inferred using the UPGMA method. The bootstrap consensus tree inferred from 250 replicates is taken to represent the evolutionary history of the taxa analyzed. Branches corresponding to partitions reproduced in less than 50% bootstrap replicates are collapsed. The percentage of replicate trees in which the associated taxa clustered together in the bootstrap test (250 replicates) is shown next to the branches. The tree is drawn to scale, with branch lengths in the same units as those of the evolutionary distances used to infer the phylogenic tree. The evolutionary distances were computed using the Maximum Composite Likelihood method and are in the units of the number of base substitutions per site. All positions containing gaps and missing data were eliminated from the dataset (Complete deletion option). There were a total of 7106 positions in the final dataset. Phylogenetic analyses were conducted in MEGA4 (9).

By identifying areas of homology among various *Borrelia* species, in combination with primer walking, we sequenced a 7231 bp genomic region comprising the *rrs* gene and the entire *rrs*-*rrl*A intergenic spacer from a human blood sample positive for *Borrelia* by microscopy (isolate HL2610, accession number: HM131216). This region comprised in the following order: the 1526 bp of the *rrs* gene and a 5705 bp region from the 16S rRNA to the 5′end of the 23S rRNA genes, containing the sequences encoding the Ala tRNA (*ala*T), the Ile tRNA (*ile*T), the adenylosuccinate lyase (*pur*B), the adenylosuccinate synthetase (*pur*A,), the methylpurine-DNA glycosylase (*mag*), the hypoxanthine-guanine phosphoribosyltransferase (*hp*t), the hydrolase (HAD superfamily) genes and a 135 bp 5′ end fragment of the 23S rRNA *(rrl*A) gene (Figure 1). Parts of this region were sequenced from seven additional isolates (accession numbers: HM161644-HM161653; HM194744-HM194759; HM194761-HM194767; HM194760; HM194726-HM194728; HM194731-HM194737; HM194729-HM194730). Alignment of all sequences revealed a low level of polymorphism among isolates in this part of the genome (Figure 1). The arrangement of the genes and their orientation were similar to those found in *B. duttonii* [16], *B. recurrentis* [16], *B. turicatae* and *B. hermsii* [16], [17], and different from the *B. burgdorferi* species [8], [16] in which a 3052-bp region separates the 16S rRNA from the 23S rRNA genes [9], [17] (Figure1). We used the sequences of this large contig to generate a phylogenic tree with other TBRF *Borrelia* for which the equivalent sequences were available. As shown in Figure 2, the Israeli *Borrelia* isolate is well separated from both Old World and New World *Borrelia* species and could be defined as a separate species from other TBRF *Borrelia* species.

### Sequencing and analysis of the *rrs-ile*T intergenic region

The *rrs-ile*T region sometimes described as intergenic spacer (IGS) has been used in several studies for the taxonomic analysis *Borrelia* species [9], [18], [19], [20]. We amplified and sequenced this region in 16 independent *B. persica* isolates from human blood and from *O. tholozani* that were collected in Israel and the West Bank. The size of this region was found to be 439 bp in all isolates tested in this work. This is the smallest IGS of known Old World TBRF *Borrelia* species [18]. The analysis of these sequences revealed three groups with nucleotide substitutions in only two positions (Table 3). The 439 bp *rrs-ile*T region contains the *ala*T gene (Figure 1), but is otherwise non-coding. Surprisingly, in spite of the non-coding nature of most of this region, the level of similarity among the studied strains was high (99.3–100%) whereas with other *Borrelia* species the level of similarity was considerably lower, ranging between 59.0–70.9% (Table 4). This observation is consistent with the low level of diversity found in IGS sequences of *B. hispanica* and *B. recurrentis* [18], [19], [21], in contrast to the high diversity found in the New World TBRF *Borrelia hermsii* [22], in Lyme disease *Borrelia* [9] and in recently deposited IGS sequences obtained (DQ768099-DQ768103, DQ768105) from Israeli human, feline and canine hosts that demonstrated high diversity even among themselves.

**Table 3:** Variable nucleotide positions and genovar definition based on the *rrs-ile*T IGS of 16 human and tickborne isolates of *B. persica* in Israel and the West Bank.

Genovars	No of strains^1^	Nucleotide at position^2^	
		5319	5377
a	5	A	A
b	10	A	C
c	1	G	A

1 List of isolates in each genovar is given in Table 1.

2 Positions of nucleotide changes are numbered according to the 7231 contig (HM131216) (this work).

**Table 4:** Coefficient of similarity between genetic loci of local *B. persica* and other TBRF *Borrelia* species.

*Borrelia* species	Coefficient of similarity of local *B. persica* genetic loci (%)				
	*rrs*	(*rrs-ileT*) IGS	*fla*B	*pur*A	*glp*Q
*B. persica* (local)	99.9–100	99.3–100	99.5–100	99.7–100	99.5–100
*B. persica* (Iran)	99.8	na	na	na	93.9
*B. recurrentis*	98.8	66.5	88.6	89.1	89.7
*B. duttonii*	99.0	62.5	88.2	88.9	89.5
*B. hispanica*	99.0	70.9	na	na	na
*B. hermsii*	98.4	59.0	86.5	85.1	86.5
*B. turicatae*	98.6	63.6	87.5	83.9	87.3

na: not available.

All local strains were included in the assessment of similarity range among *B. persica* isolates studied in this work. We used the sequences of *B. persica* strain HL2610 (relevant accession numbers are listed in materials and methods) for comparison with other TBRF *Borrelia* available homologous genes: *B. persica* Iran *rrs* (U42297), *glp*Q of *B. persica* Iran (EU914143), *B. hispanica rrs* (U42294), *B. hispanica rrs-ileT* IGS (FJ827590), *B. recurrentis* complete genome (CP000993), *B. duttonii* complete genome (CP000976), *B. hermsii* complete genome (CP000048) and *B. turicatae* complete genome (CP000049). Vector NTI advance 11 software was used for sequence alignments.

**Figure 3:**
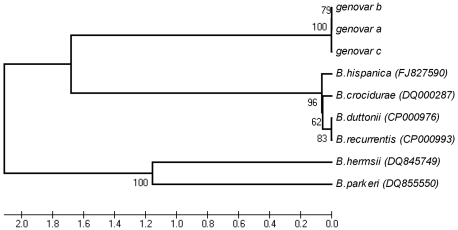
Phylogenic tree based on *rrs-ile*T spacer (IGS) sequences. The *rrs-ile*T spacer (IGS) sequences for 16 independent isolates from Israel and the West Bank belonging to genovars a to c were compared to IGS sequences from other *Borrelia* species (accession numbers are given in parentheses). The isolates in each genovar are listed in Table 1. The phylogenic tree was inferred using the UPGMA method as described in Figure 2. All positions containing gaps and missing data were eliminated from the dataset (Complete deletion option). There were a total of 349 positions in the final dataset.

Alignment with other *Borrelia* sequences showed that the IGS locus was especially discriminatory between the local *Borrelia* isolates and the Old or New World TBRF *Borrelia* species (Figure 3). The 439 bp *rrs-ile*T region sequence is not available for the prototype Iranian strains but, as in the case for the entire *rrs-rrl*A intergenic spacer, the comparison of this sequence with other *Borrelia* species clearly showed that all Israeli and Palestinian isolates formed a unique cluster separated from all other TBRF *Borrelia* species (Figure 3).

### Sequencing and analysis of the 16S rRNA gene (*rrs*)

Although analysis of the 7231 bp fragment clearly classifies the HL2610 isolate as an individual species when compared to other TBRF *Borrelia* species, this complete sequence was not reported for the Iranian reference *B. persica* strain [7] and therefore could not be used for the definitive classification of the isolate HL2610 as *B. persica*.

For speciation of the local isolates described in this work, we compared the complete sequence of the *rrs* gene from the reference *B. persica* originally isolated in Iran (U42297) [7] and the partial *rrs* gene sequence of an independent Iranian *B. persica* strain IRbp1 (U914141) to *rrs* sequences of local isolates (Table 1). The complete 16S rRNA genes of three human TBRF *Borrelia* isolates (HL2610, H1254, H1039) were sequenced (Table 5). The resulting 16S rRNA sequences were aligned with that of the prototype *B. persica* Iranian strain (U42297) [7]. Positions of polymorphism between Israeli and the Iranian strain are summarized in Table 3 (C to T at position 1435, A to G at position 1443, a C insertion at position 371 in all the sequences, and in the case of strain H2610 an additional A to G at position 625). The C insertion at position 371 has been also described for both Old and New World TBRF *Borrelia* species (*B. duttonii*: U42288, AF107364; *B. hispanica*: U42294, DQ057988; *B. hermsii*: U42292, CP000048; *B. turicatae*: U42299, CP000049). The coefficients of similarity between the local and Iranian strains were very high (99.8%) suggesting that these isolates constitute a single species, different from other TBRF *Borrelia* which showed coefficients of similarity ranging between 99.1–98.4% (Table 4). As expected, the coefficient of similarity with *B. burgdorferi* was much lower (96%). These results classify the Israeli isolates as *B. persica*. The complete prototype *rrs* sequence was used by Ras et al. [7] for the classification of *B. persica* in relationship to other TBRF *Borrelia* species. Given the low discriminatory power of this conserved gene, the authors concluded that the classification of *B. persica* as an independent species is relevant but not fully satisfactory [3], [7]. Stackebrandt and Ebers suggested that a cutoff range of 98.7–99.0% in the 16S rRNA gene homology is appropriate for species differentiation within a genus [23]. According to these criteria, the data presented here clearly differentiate *B. persica* from other TBRF *Borrelia* species and definitively allow the classification of the agent responsible for TBRF in our region as *B. persica*.

**Table 5:** Variability and genovar distribution based on the 16S rRNA gene (*rrs*) sequences of *B. persica* in Israel and Iran.

Location	Genovar	Number of strains^1^	Nucleotides at position^2^			
			371	**625**	1435	1443
Israel	R1	7(2)	C insertion	**G**	C	A
Israel	R2	3(1)	C insertion	**A**	C	A
Iran	R1	2(1)	no C insertion	**G**	T	G

The accession numbers of all *rrs* sequences of Israeli isolates, complete and partial, are listed in materials and methods. Accession numbers of the Iranian strains are U42297 (complete sequence) and EU914141 (partial sequence).

1 The number in parenthesis indicates the number of isolates in each genovar for which the complete *rrs* sequences are available.

2 Numbering of the nucleotide positions is according to the complete *rrs* sequence of the R1 strain H1039 (HM161645). Bold letters indicate the nucleotide changes that define the genovars.

The *rrs* sequences of the three Israeli isolates, originating from different locations were identical except for one mismatch (A to G at position 625) (Table 5). Sequence analysis of a 498 bp fragment of the *rrs* gene comprising the position 625 (amplified by primers 16S1F and 16S7R) from seven additional samples confirmed the existence of two distinct *B. persica* genovars based on the *rrs* sequence: genovar R1 with a G and genovar R2 with an A at position 625 (Table 5). Partial sequences of the *rrs* gene from previously published Israeli isolates (DQ207600-DQ207603, DQ768104, AY763792) showed a similar A to G distribution. The *B. persica* strains isolated in Iran, the prototype strain (U42297) and the partial IRbp1 strain (EU914141) have a G at position 625 thus belonging to genovar R1.

The distribution of the local isolates between two genovars based on the A to G modification in the *rrs* gene places *B. persica* in a unique phylogenic position. The analysis of the available *rrs* sequences of TBRF *Borrelia* indicates that the presence of A or G at this position discriminates between Old World (*B. recurrentis*, *B. duttonii*, *B. crocidurae and B. hispanica*) and New World (*B. hermsii*, *B. parkeri*, *B. turicatae*) TBRF *Borrelia*, so that a G is characteristic of New World species whereas A is a signature of the Old World TBRF *Borrelia*. *B. persica* is the only species were both possibilities are present.

**Figure 4:**
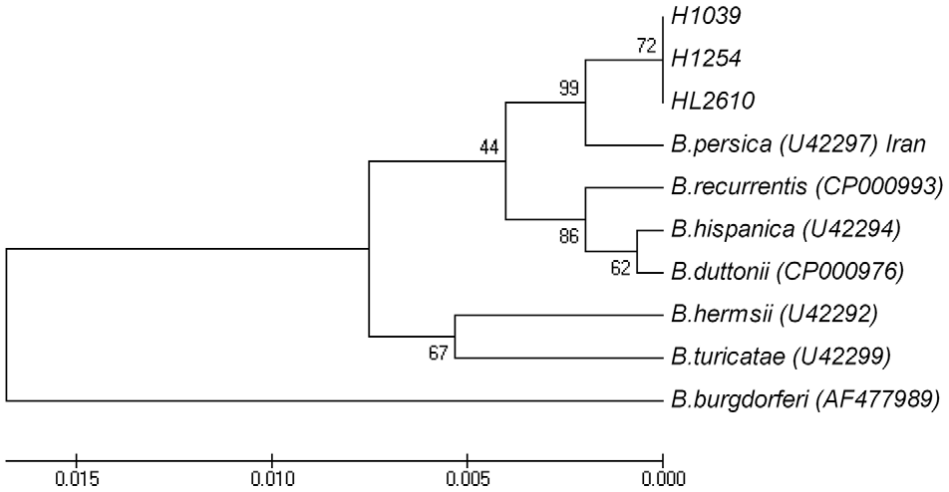
Phylogenic tree based on *rrs* sequences. The complete *rrs* sequences of *B. persica* isolates in Israel were compared to *rrs* sequences from *B. persica* (Iran) and other *Borrelia* species (accession numbers are given in parentheses). The phylogenic tree was inferred using the UPGMA method. Parameters were as described in Figure 2. All positions containing gaps and missing data were eliminated from the dataset (Complete deletion option). There were a total of 1522 positions in the final dataset.

The relationship among the TBRF species is also reflected in the phylogenic tree presented in Figure 4. The analyzed *Borrelia* species were separated into two clusters, one including the New World species *B. turicatae* and *B. hermsii* and, the second dividing into two branches that included on the one hand the African strains *B. hispanica*, *B. duttonii* and *B. recurrentis* and on the other hand all Israeli isolates that grouped together with the reference Iranian *B. persica* strain.

### Sequencing and analysis of the *pur*A gene

**Figure 5:**
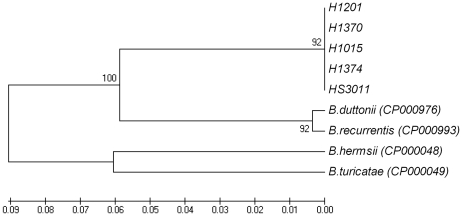
Phylogenetic tree based on *pur*A nucleotide sequences. The complete *pur*A sequences of *B. persica* isolates in Israel and the West Bank were compared to *pur*A sequences from other *Borrelia* species (accession number are given in parentheses). The Phylogenic tree was inferred using the UPGMA method. Parameters were as described in Figure 2. All positions containing gaps and missing data were eliminated from the dataset (Complete deletion option). There were a total of 1284 positions in the final dataset.

Among the genes in the large 7231 bp contig, the hypoxanthine-guanine phosphoribosyltransferase (*hpt*), adenylosuccinate lyase (*pur*B) and adenylosuccinate synthase (*pur*A) belong to a group of six open reading frames found in the TBRF *Borrelia* species but not in *B. burgdorferi* [24]. The 1284 bp sequence of the *pur*A gene was amplified and sequenced. Analysis of sequences from eight *B. persica* isolates, seven from human hosts and one from *O. tholozani* revealed the presence of 4 genovars reflecting five base pair substitutions, two of which result in amino acid substitutions Ser to Leu in genovar P3 and Ser to Pro in genovar P4) (Table 6). The data obtained from the analysis of *pur*A sequences confirmed that all the local strains belonged to a single phylogenetic cluster (Figure 5).

**Table 6:** Variable nucleotide positions and genovar definition based on the *pur*A gene of 8 human and tickborne isolates of *B. persica* in Israel and the West Bank.

Genovar	Number of strains^1^	Nucleotide at position^2^				
		2417	2444	2545	2615	3003
P1	4	T	C	T	A	T
P2	1	C	A	T	G	T
P3	2	T	C	C(Ser>Leu)	A	T
P4	1	nd	nd	nd	A	C(Ser>Pro)

nd: not done.

1 List of isolates in each genovar is given in Table 1.

2 Positions of nucleotide changes are numbered according to the 7231 contig (HM131216) (this work). Where relevant, the resulting amino acid modification is shown in parenthesis.

The coefficient of similarity between the *pur*A gene sequences of the eight isolates from ticks or human varied from 99.7% to 100% whereas the coefficient of similarity with the *pur*A of other TBRF *Borrelia* species varied from 83.9% to 89.1% (Table 4) confirming once more the status of *B. persica* as a distinct TBRF *Borrelia* species.

### Sequence analysis of the flagellin gene (*fla*B)

The sequence of *fla*B and the sequences of the *rrs-rrl*A intergenic region are often used for molecular epidemiology of *Borrelia* ssp. [9], [18], [25] but unfortunately are not available for the strains from Iran. Analysis of Israeli strains based on the *fla*B gene has been described before and three distinct genovars (I to III) were defined [2]. Typing is performed by amplifying and sequencing a 750 bp fragment of the *fla*B gene delineated by primers BOR1 and BOR2 (Table 2). The sequencing of the partial *fla*B genes of 12 human and ticks samples originating from Israel and the Palestinian Authority showed that the isolates belonged either to type I or to type II (Table 1) described previously [2]. The level of similarity (99.0–100%) and modifications found in this study were identical to those described by Assous et al. [2].

Phylogenic analysis based on the partial *fla*B sequences (data not shown) confirmed that all local isolates from human as well as tick samples formed a unique cluster, different from the New World and Old World clusters [3].

### Sequence analysis of the glycerophosphodiester phosphodiesterase (*glp*Q) gene

Similarly to the *pur*A gene, the presence of the *glp*Q gene differentiates TBRF *Borrelia* from Lyme disease *Borrelia* species that lack these genes [24], [26], [27], [28].

**Figure 6:**
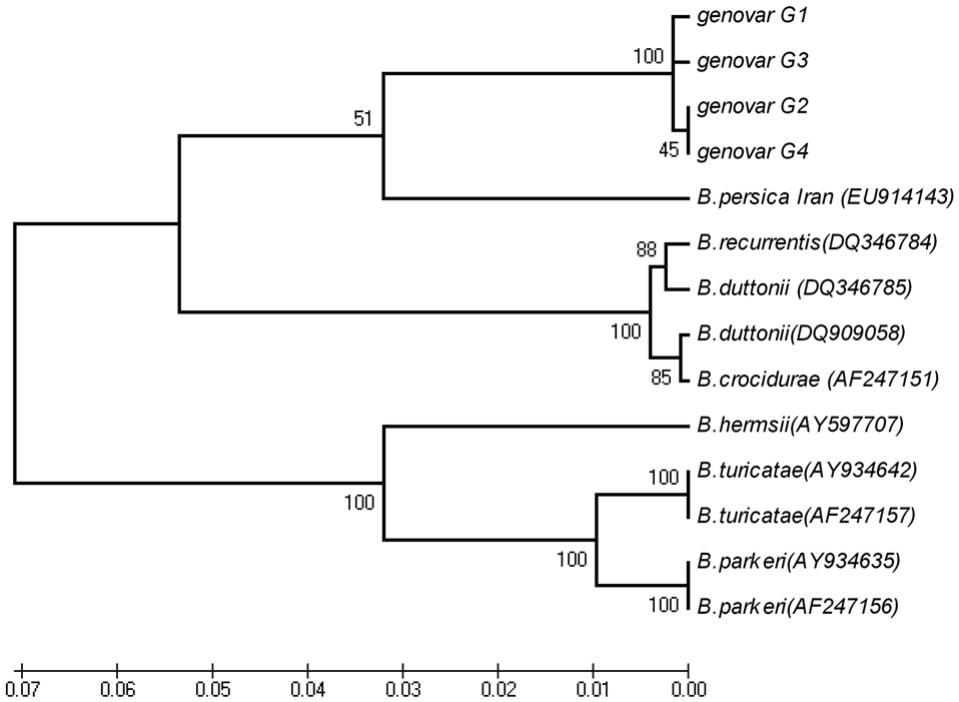
Phylogenic tree based on *glp*Q nucleotide sequences. *glp*Q sequences of 18 independent isolates from Israel and the West Bank belonging to genovars G1 to G4 were compared to *glp*Q sequences from other *Borrelia* species (accession numbers are given in parentheses). The isolates in each genovar are listed in Table 1. The phylogenic tree was inferred using the UPGMA method as described in Figure 2. All positions containing gaps and missing data were eliminated from the dataset (Complete deletion option). There were a total of 637 positions in the final dataset.

We amplified and sequenced an 813 bp fragment of the *glp*Q gene from 18 samples isolated in various locations in Israel and the West Bank (Table 1). Polymorphism of the *glp*Q gene among the 18 samples amplified by PCR is shown in Table 7 with substitutions at position 181 (G to A), 472 (C to T) and 613 (G to T resulting in a change of amino acid Ala to Ser) creating four different genovars (G1 to G4). Recently the sequence of a 668 bp fragment of the *glp*Q gene from an Iranian strain (IRbp1) was published (accession number EU914143). Although this strain is different from the Iranian reference strain used for sequencing of the 16S rRNA gene, it allows the analysis of phylogenic relationship between Israeli and Iranian *Borrelia* based on this locus. The coefficient of similarity among the sequences of the different isolates from ticks or human whether in the West Bank or in Israel varied from 99.5 to 100%.The of similarity with *glp*Q sequence from Iran was only 93.8% (Table 4). However, the coefficient of similarity with the *glp*Q sequences of other TBRF *Borrelia* species was considerably lower and varied from 89.7% to 86.5% (Table 4). The phylogenic tree generated using these sequences (Figure 6) showed that isolates in Israel/Palestinian Authority formed a cluster well separated from Old World and New World *Borrelia* spp. strengthening the classification of the isolates in Israel and in the Palestinian Authority as *B. persica*. The Iranian strain represented a separate branch, probably reflecting an independent clonal evolution of a subspecies of *B. persica*; nevertheless this branch was very close to the branch containing all the strains described in this work and the two branches were clearly separated from other *Borrelia* species (Figure 6).

**Table 7:** Variable nucleotide positions and genovar definition based on the sequences of the *glp*Q gene of 18 human and tick-borne isolates of *B. persica* in Israel and the West Bank.

Genovar^1^	No. of strains	Nucleotide at position^2^		
		181	472	613
G1	3	G	C	G
G2	11	A	T	G
G3	3	G	T	G
G4	1	A	T	T (Ala>Ser)

1 List of isolates for each genovar is given in Table 1.

2 Positions of nucleotide changes are numbered according to the *glp*Q sequence of isolate H1015 (HM161654) (this work). Where relevant, the resulting amino acid modification is shown in parenthesis.

A partial sequence of the *glp*Q gene from *B. persica* isolated in Israel was published before [4]. Its coefficient of similarity to the *glp*Q sequences in our work is only 91.1%. Surprisingly that *glp*Q sequence showed a very high degree of similarity (98.4%) with the *glp*Q gene of *B. duttonii* with near identity (99.0%) at the protein level. The partial sequence of the *rrs* gene of this isolate suggests that it belongs to genovar R1. Such a *glp*Q variant is not represented among the isolates in our collection and may indicate that the diversity among *B. persica* strains in the Middle East is larger than suggested before.

### Multi locus sequence analysis (MLSA)

**Figure 7:**
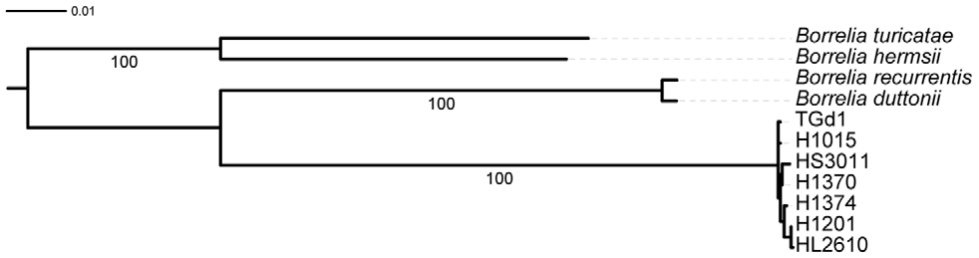
Phylogenetic tree based on the concatenated alignments of *pur*A, *glp*Q, *fla*B and *rrs-ile*T IGS nucleotide sequences. The tree was inferred using the PHYML program. The percentage of trees in which the associated taxa clustered together in the bootstrap test (100 replicates) is shown next to the branches if higher than 80%. The tree was arbitrarily rooted at midpoint.

To definitively confirm the speciation of the Israeli and Palestinian authority *Borrelia* isolates, MLSA was performed on seven isolates originating from human and tick samples and belonging to both 16S rRNA genovars (Figure 7). The MLSA study was based on genes of different loci both coding (*pur*A, *fla*B and *glp*Q) and the non-coding (*rrs-ile*T IGS) sequences. The alignment of the sequences for each of the loci reveals a low level of diversity among the isolates (Table 4). A concatenated sequence based on these genes was used for a phylogenic analysis with homologous loci of other TBRF *Borrelia* species. The seven independent local isolates formed a unique cluster separated from all other species (Figure 7).

Sequencing of six or more genes from different loci allows a multilocus sequence analysis (MLSA) that can define bacterial species without sequencing a whole genome or performing DNA-DNA hybridizations [29]. Average nucleotide identity of 95% or more defines a species when using MLSA [29]. Although we used only four loci (besides the 16S rRNA gene), our results show that the average nucleotide identity among the local *B. persica* isolates was higher than 99% whereas with other *Borrelia* species, the identity was less than 90% (Table 4), confirming the status of species for *B. persica* and classifying the Israeli and Palestinian isolates as *B. persica* independently of the 16S rRNA data presented above.

In this work we were able to classify the human and tick TBRF *Borrelia* isolates originating from the Palestinian Authority and from Israel as a single defined species *B. persica*. Indeed, our data confirm for the first time, that *B. persica* is the infectious agent of TBRF in Israel and the Palestinian Authority. *B. persica* forms a distinct phylogenetic group that is located between the TBRF *Borrelia* species of the Old World and the New World. Differences in the *fla*B, *glp*Q and IGS loci allowed for more specific typing of the *B. persica* strains, however there were no noticeable differences between human and tick isolates confirming the co-speciation between *B. persica* and the vector *O. tholozani*. Further studies are needed to discover the reservoir of the agent of TBRF in our region.

The data generated in this work are now available for the design of specific primers and probes for comprehensive epidemiological studies and evaluation of diagnostic assays that will improve the detection of the disease in our region.
